# Suspected Virus-Inducing Severe Acute Respiratory Distress Syndrome Treated by Multimodal Therapy Including Extracorporeal Membrane Oxygenation and Immune Modulation Therapy

**DOI:** 10.7759/cureus.8768

**Published:** 2020-06-22

**Authors:** Saya Ikegami, Kei Jitsuiki, Hiroki Nagasawa, Ryota Nishio, Youichi Yanagawa

**Affiliations:** 1 Acute Critical Care Medicine, Juntendo University Shizuoka Hospital, Izunokuni, JPN

**Keywords:** extracorporeal membrane oxygenation, immune modulation therapy, acute respiratory distress syndrome

## Abstract

A 44-year-old man who had been feeling general fatigue was found in an unconscious state on the same day. He had no remarkable medical history. On arrival at the hospital, his Glasgow Coma Scale was E1V2M3; he had tachycardia and hypertension, was afebrile, and in a severe hypoxic state. His PaO_2_/FiO_2_ (P/F) was under 100, even with tracheal intubation with 100% oxygen. Chest X-ray and CT revealed a bilateral ground-glass appearance with consolidation. Cardiac echo initially showed hyper-dynamic wall motion. The main results of a blood analysis suggested an acute inflammatory reaction, rhabdomyolysis, and pancreatitis. The microscopic findings of sputum and a rapid test for bacterial and viral infections were all negative. As he showed deterioration of P/F, venovenous extracorporeal membrane oxygenation (ECMO) was started. He also showed hypotension and therefore underwent vasopressor and steroid administration. Due to concerns of pneumonia, he received meropenem and azithromycin in addition to the infusion of γ-globulin and glycyrrhizin. The results of a COVID-19 test, culture of sputum, and collagen disease test were all negative. The serum virus neutralization assay as a serological test for Coxsackievirus B4 showed a four-fold increase in titer. The multimodal therapy mentioned above resulted in the improvement of his general condition, including acute respiratory distress syndrome (ARDS). In this report, we discuss the benefits of ECMO and immune modulation therapy in the treatment of severe ARDS.

## Introduction

Acute respiratory distress syndrome (ARDS) is a common cause of respiratory failure in critically ill patients and is defined by the acute onset of non-cardiogenic pulmonary edema, hypoxemia, and the need for mechanical ventilation [1-4]. The pathology of ARDS is diffuse alveolar damage, such as the rapid development of capillary congestion, atelectasis, intraalveolar hemorrhaging, and alveolar edema, followed days later by hyaline-membrane formation, epithelial-cell hyperplasia, and interstitial edema [3]. ARDS occurs most often in the setting of pneumonia, sepsis, aspiration of gastric contents, or severe trauma and is present in roughly 10% of all patients in intensive-care units worldwide [4]. Although much progress has been made in improving supportive care for ARDS, effective pharmacological therapies have not yet been identified, and mortality remains high at 30%-40% in most studies [4].

We report a case of suspected virus-inducing severe ARDS treated by multimodal therapy including extracorporeal membrane oxygenation (ECMO) and immune modulation therapy that led to a favorable outcome for the patient.

## Case presentation

A 44-year-old man felt generalized fatigue and took the day off from work. His son called him on the same day, but he did not respond. When the son visited his house, he found the patient unconscious and called an ambulance. He had no remarkable medical history. He was a never‐smoker and drank 20 g of ethanol per day. He worked as a truck driver and lived with his only son after his divorce. His work zone was not located in any of the districts that were reported to have COVID-19 infections.

When the emergency medical technicians checked him, he had a tonic convulsive posture with severe hypoxia, and he was transported to our hospital under bag-valve-mask ventilation with high-concentration oxygen. On arrival, his Glasgow Coma Scale was E1V2M3. A physical examination revealed the following findings: blood pressure of 174/130 mmHg; heart rate of 140 beats per minute; a respiratory rate of 30 breaths per minute; SpO_2 _of 75% under room 15 L per minute of oxygen; and body temperature of 36.9 °C. A venous route was immediately secured, followed by endotracheal intubation.

**Figure 1:**
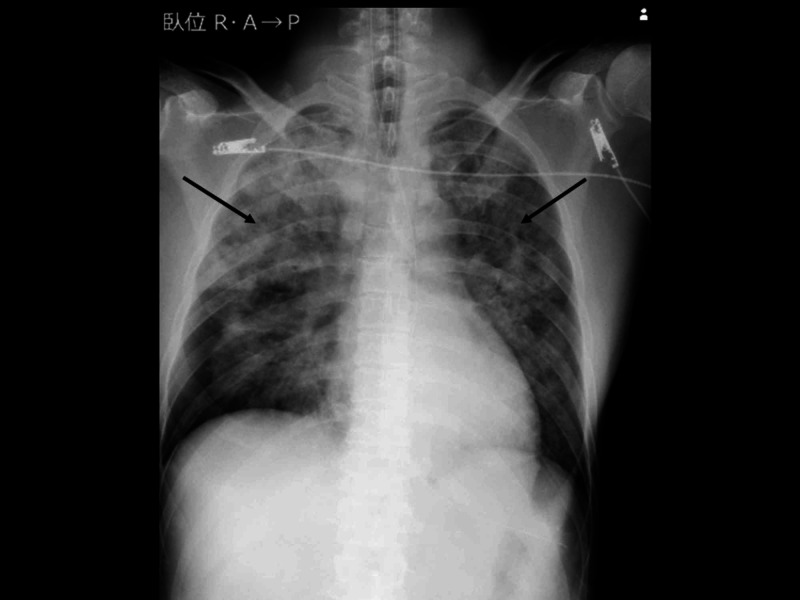
Chest X-ray on arrival The image shows a bilateral ground-glass appearance (arrow)

An arterial gas analysis revealed the following findings: pH: 7.092; PCO_2_: 54.2 mmHg; PO_2_: 54.5 mmHg; base excess -15.0 mmol/L; and lactate: 6 mmol/L. Electrocardiography revealed sinus tachycardia. A chest X-ray revealed a bilateral ground-glass appearance (Figure 1).

**Figure 2:**
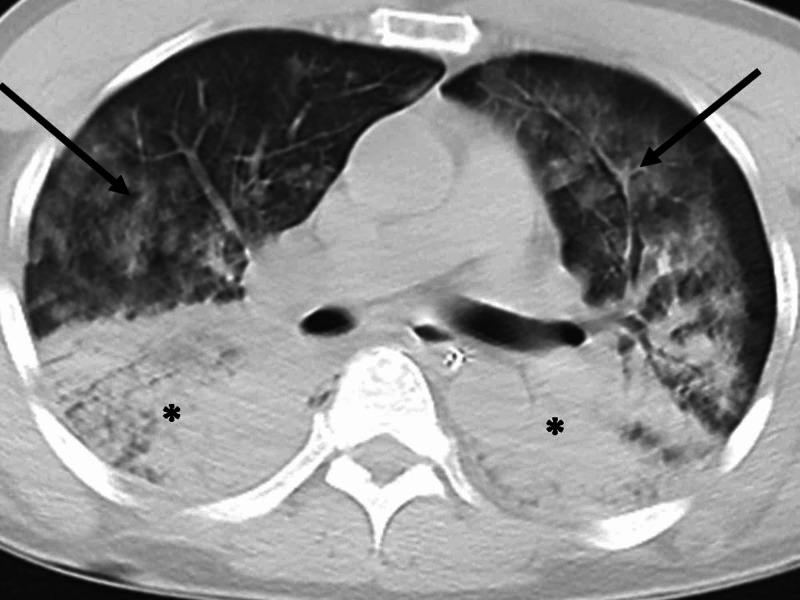
CT on arrival The image shows a bilateral ground-glass appearance in the ventral lung fields (arrow) and bilateral consolidation in the dorsal lung fields (asterisks) CT: computed tomography

Cardiac echo showed hyper-dynamic left-ventricular wall motion. Whole-body CT revealed a bilateral ground-glass appearance in the ventral lung fields and bilateral consolidation in the dorsal lung fields (Figure 2).

The pancreas was normal. The main results of a blood analysis were as follows: WBC count: 23,400/μL (neutrophil 87%, lymphocyte 6%, monocyte 6%); hemoglobin: 16.5 g/dL; platelet count: 22.0×10^4^/μL; total protein: 7.0 g/dL; albumin: 4.3 g/dL; glucose: 177 mg/dL; HbA_1_C: 5.5%; total bilirubin: 1.5 mg/dL; aspartate aminotransferase: 322 IU/L; alanine aminotransferase: 79 IU/L; lactate dehydrogenase: 1,108 IU/L; blood urea nitrogen: 7.4 mg/dL; creatinine: 0.51 mg/dL; amylase: 413 (pancreas 76%) IU/L; creatine phosphokinase (CK): 44,139 IU/L; sodium: 110 mEq/L; potassium: 4.2 mEq/L; chloride: 79 mEq/L; brain natriuretic peptide: 760.1 pg/mL; C-reactive protein: 22.2 mg/dL: prothrombin time international normalized ratio: 1.02; activated partial thromboplastin time: 34.6 (26.6) seconds; fibrinogen: 242 mg/dL; D-dimer: 1.5 μg/mL; human immunodeficiency virus (HIV) antibody: negative; pneumococcal urinary antigen test: negative; legionella urinary antigen test: negative; rapid influenza diagnostic test: negative; microscopic finding of sputum: negative: and urine drug screening test: negative. Later, β-d glucan, rheumatoid factor, and anti-neutrophil cytoplasmic antibody were all found to be negative. He received a tentative diagnosis of pneumonia of unknown causes accompanying severe ARDS, rhabdomyolysis, and pancreatitis.

As he showed deterioration of PaO_2_ [PaO_2_/FiO_2_ (P/F) = 50] and a Murray score of 3.2, he underwent mechanical ventilation under 1.0 FiO_2_ and 10 cmH_2_O with positive end-expiratory pressure (PEEP) and received indwelling venovenous ECMO (MERA centrifugal blood pump system HAS‐CFP; MERA NHP exelung NSH‐R HPO‐23WH‐C; Senko Medical Instruments, Tokyo, Japan) with the right jugular vein (return side, 14 Fr) and right femoral vein (drainage side, 20 Fr) as the exit. As this event occurred during the night shift, we did not attempt supine therapy before introducing venovenous ECMO. He also showed hypotension and therefore underwent infusion of noradrenalin followed by vasopressin and 200 mg of hydrocortisone. Due to concerns of pneumonia, he received 1.5 g of meropenem and 500 mg of azithromycin in addition to an infusion of 5 g of γ-globulin and 40 ml of glycyrrhizin as antibacterial and antivirus treatments respectively.

After the induction of ECMO, the mode of mechanical ventilation was changed to a lung rest setting (0.25 FiO_2_ and 5 cmH_2_O with PEEP). Additional cardiac echo showed diffuse hypokinesis with a 30% ejection fraction, and troponin T became positive [207 pg/mL (normal range: <14 pg/mL)] on the first hospital day, and so he was diagnosed with myocarditis as a complication. On the second day, his blood pressure increased, resulting in a reduction in the vasopressor administration. His P/F remained under 100. On the third day, inflammatory data remained high, and hence azithromycin was replaced with levofloxacin. However, this led to skin reddening and he was switched back to azithromycin. A complication of anemia, thrombocytopenia, and coagulopathy required blood transfusion. A polymerase chain reaction (PCR) test for COVID-19 using sputum through the endotracheal tube without bronchoalveolar lavage (performed twice) and initial cultures of sputum, urine, and blood were all negative.

**Figure 3:**
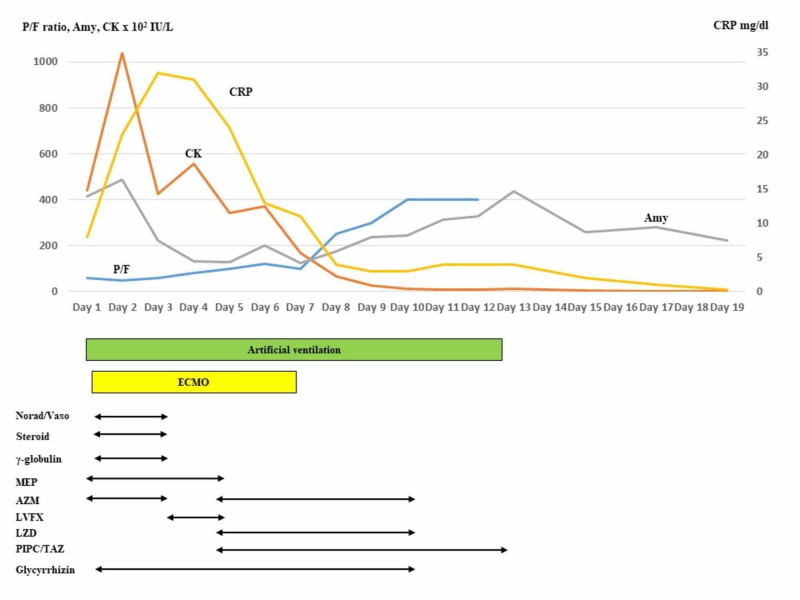
The time course of PaO2/FiO2 (P/F), the main laboratory data, and the treatment. P/F was improved by multimodal therapy Amy: amylase; CK: creatine phosphokinase; CRP: C-reactive protein; Norad/Vaso: noradrenaline/vasopressin; MEP: meropenem; AZM: azithromycin; LVFX: levofloxacin; LZD: linezolid; PIPC/TAZ: piperacillin/tazobactam

On the fifth day, the inflammatory data remained moderate, and hence meropenem was replaced with linezolid and piperacillin/tazobactam. On the same day, in an attempt to withdraw ECMO, the mechanical ventilation setting was changed from 5 to 10 cmH_2_O for PEEP and from 0.25 to 0.4 for FiO_2_. This resulted in the P/F increasing to over 100. After the circulation flow of ECMO was reduced from 3 to 1 L/minute, the P/F remained over 100; hence ECMO was ceased, and the cannulations were removed (Figure 3).

**Figure 4:**
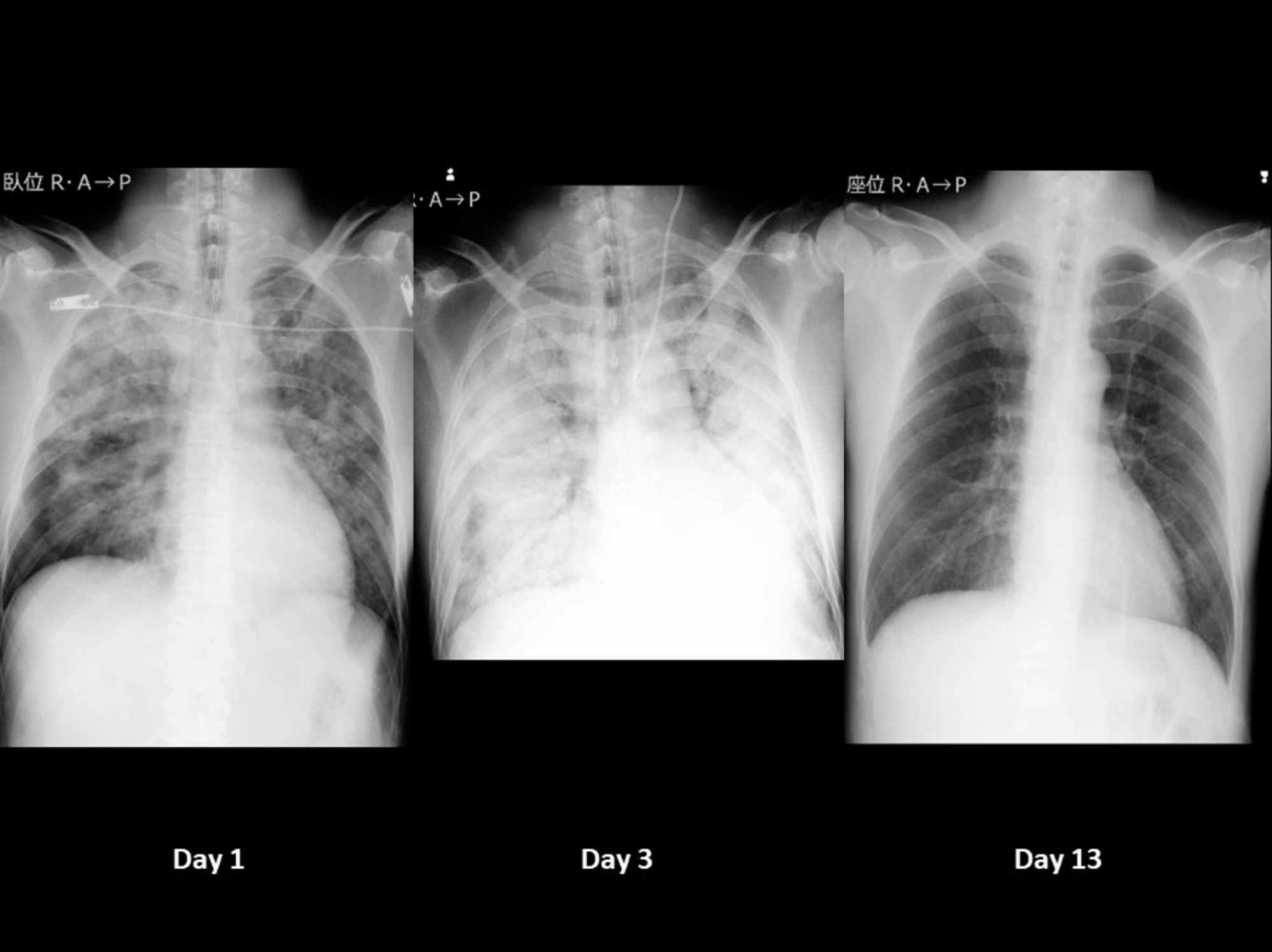
Time course of chest X-ray The patient was managed by extracorporeal membrane oxygenation and mechanical ventilation with the lung rest setting on the third day and was withdrawn from mechanical ventilation on the 13th day Day 1 (left) shows bilateral ground-glass appearances. Day 3 (middle) shows the deterioration of bilateral radiolucency. Day 13 (right) shows clear lung fields

As the patient was thought to require long-term mechanical ventilation, he underwent tracheostomy on the eighth hospital day. Sedative administration was ceased, and a negative water balance was targeted by limiting the infusion volume and the use of diuretics. He showed a transient decrease in his P/F by the formation of atelectasis due to bloody sputum; however, the average P/F improved day by day. On the 12th hospital day, his P/F exceeded 300 under 5 cmH_2_O for PEEP and 0.25 FiO_2_; hence mechanical ventilation was ceased (Figure 4).

He recovered his ability to excrete sputum by himself, and tracheal cannulation was removed on the 13th hospital day. Even after ceasing all drugs for lung and inflammation, his pneumonia, rhabdomyolysis, and pancreatitis did not recur. He was discharged on the 22nd day on foot. The troponin T level remained high (334 pg/mL) even after the CK level normalized, so he was followed up as an outpatient. The serum virus neutralization assay performed as a serological test using pair serum samples with a more than two-week interval for Coxsackievirus B4 showed a four-fold increase in titer (from x64 to x256). Finally, the troponin T level returned to the normal range and showed no subsequent complications.

## Discussion

Risk factors of direct lung injury involving ARDS include pneumonia (bacterial, viral, fungal, or opportunistic), aspiration of gastric contents, pulmonary contusion, inhalation injury, and near-drowning, while those of indirect lung injury involving ARDS include sepsis (non-pulmonary source), non-thoracic trauma or hemorrhagic shock, pancreatitis, major burn injury, drug overdose, transfusion of blood products, cardiopulmonary bypass, reperfusion edema after lung transplantation, and embolectomy [3,4]. The risk factor in the present case was unspecified pneumonia, and an unspecified virus was considered the most likely cause based on the negative results of all cultures, β-D glucan, and rapid test for bacteria and influenza. Concerning COVID-19, while an outbreak had been reported in Tokyo at the time, our patient did not live in a COVID-19-infected district; in addition, two tests for COVID-19 were negative, and CT showed subpleural sparing with a ground-glass appearance, which is not common for COVID-19. Accordingly, the possibility of COVID-19-related pneumonia was considered to be low. Based on our investigations for virus infection, we suspect that Coxsackievirus B4 might have been the responsible virus. However, there are few reports concerning Coxsackievirus B infection with pulmonary involvement, and there have been no reports of ARDS induced by Coxsackievirus B4 [5]. Accordingly, this might be the first case report of severe ARDS in an adult induced by Coxsackievirus B4 infection. As we did not perform a direct examination of a pulmonary specimen, the possibility of co-infection with multiple viruses or reactivation of Coxsackievirus B4 cannot be excluded.

There are two main treatments for ARDS: treating the main cause of ARDS or offering supportive therapy for the severely injured lungs. Supportive therapy involves oxygen and mechanical ventilation. According to the Berlin definition, severe ARDS requires mechanical ventilation with a high PEEP and a high concentration of oxygen. When conventional mechanical ventilation fails to improve the arterial oxygenation and/or eliminate carbon dioxide, ECMO is indicated. Another indication is circulatory and/or cardiac failure. The conventional ventilation mode can cause ventilator-induced lung injury, such as volutrauma, atelectrauma, and/or biotrauma. In addition, the continuous inspiration of high-concentration oxygen can also injure the lungs. The induction of ECMO helps avoid such mechanical- and oxygen-induced lung injuries by using the lung rest setting, allowing patients time to recover from their lung injuries [6-8]. As the present case also showed marked hypoxia despite mechanical ventilation with a high concentration of oxygen and high PEEP, ECMO was introduced, and the lung rest setting was selected.

The severity of ARDS depends on the amount of etiologic substances with corresponding immune reactions, the duration of the appearance of specific immune cells, and the repertoire of specific immune cells that control the substances. Therefore, treatment with systemic immune modulators (corticosteroids and/or intravenous immunoglobulin) as soon as possible may reduce aberrant immune responses in the early stage of ARDS [9]. The results from clinical trials have often been controversial; however, the administration of steroids may shorten the duration of mechanical ventilation, duration of hospitalization, and improve oxygenation, probably because of the wide spectrum of potentially desirable effects, including anti-inflammatory, antioxidant, pulmonary vasodilator, and anti-edematous [3,4,10]. Lee et al. noted that early systemic immune modulators (corticosteroids and/or intravenous immunoglobulin) along with antibiotics or antivirals could halt the progression of pneumonia and induce a rapid recovery of pulmonary lesions in patients with ARDS [9]. Furthermore, macrolides also induce a broad range of immunological mechanisms that result in immunomodulatory effects; hence macrolide therapy can also help reduce mortality in patients with ARDS [11]. Accordingly, the variety of supportive therapies offered for ARDS may explain the favorable outcome in the present case.

Viral pneumonia was thought to be the most likely cause of ARDS in the present case. Respiratory viruses are a common cause of severe pneumonia and ARDS in adults [3,12]. Initially, the present case was suspected of having COVID-19 infection, but this diagnosis was not supported by PCR performed twice. The percentage of patients with ARDS for which no causative organism has been identified despite bronchoalveolar lavage or PCR testing remains high (>50-60%) [4]. Although antiviral therapy is available for some respiratory viral infections, most viruses do not have any specific treatment. One of the antivirus therapies used in the present case was glycyrrhizin. Glycyrrhiza glabra roots contain glycyrrhizic acid (glycyrrhizin), which is effective against viruses [13]. Glycyrrhizin inhibits the growth and cytopathology of several unrelated DNA and RNA viruses while not affecting human cell activity or their ability to replicate [13]. Glycyrrhizin is therefore now applied in the treatment of a variety of viral infections [14]. Traditional Chinese medicines such as glycyrrhizin may also be effective against COVID-19 infection [15]. In addition, macrolide and γ-globulin can also exert an antiviral effect [16]. These unspecific antiviral therapies may be useful treatments for the main cause of ARDS, which may have resulted in the favorable outcome obtained in the present case.

The present case showed pneumonia, pancreatitis, rhabdomyolysis, and myocarditis. The involvement of two pathogens (Salmonella typhi and Mycoplasma) that have been reported to accompany such complications was not found in the present case [17,18]. However, cases of pneumonia, rhabdomyolysis, myocarditis, and pancreatitis induced by Coxsackievirus B4 have been reported [19-20]. Accordingly, this virus may have been the causative pathogen of these complications in the present case.

## Conclusions

We presented a case of suspected virus-inducing severe ARDS that was treated by multimodal therapy including ECMO and immune modulation therapy. The wide range of supportive therapies and unspecific antiviral therapies offered for ARDS may have resulted in the favorable outcome obtained in the present case.
